# Feasibility of transthoracic esophagectomy with a next-generation surgical robot

**DOI:** 10.1038/s41598-022-21323-z

**Published:** 2022-10-26

**Authors:** Shailesh Puntambekar, Suyog Bharambe, Swapnil Pawar, Mihir Chitale, Mangesh Panse

**Affiliations:** Galaxy Care Laparoscopy Institute, Galaxy Care Hospital, Pune, Maharashtra India

**Keywords:** Gastrointestinal system, Oesophagus, Surgery

## Abstract

Robot-assisted minimal access surgery (MAS), compared with conventional MAS, has shown a number of benefits across several therapeutic indications but its use for transthoracic esophagectomy (TTE) requires further evaluation. Here, we report the first-in-human series of major esophageal resections performed using a next-generation tele-operated robotic surgical system in a single center. Robot-assisted TTE was performed using the Versius Surgical System by a single surgeon to assess the robotic system’s ability to achieve tumor clearance (measured by R0 resection rates) whilst reducing anastomotic leakage rates. Intra- and post-operative outcomes such as median operative time, length of hospitalization, intra-operative blood loss, and the number of complications were also assessed. Fifty-seven patients underwent robot-assisted TTE between August 2019 and June 2021. All procedures were completed successfully with no unplanned conversions to alternative surgical methods. Estimated blood loss was minimal, and no adverse events, complications or deaths were reported. Our experience with the Versius Surgical System demonstrates its safe adoption and implementation for TTE.

## Introduction

Compared to open surgery, minimal access surgery (MAS) can minimize intra-operative blood loss, post-operative pneumonia, length of hospital stay, and improve 1-year survival rates in patients requiring esophagectomy^1,2^. Aiming to reduce invasiveness and morbidity, MAS has been implemented worldwide and is often preferable to open esophagectomy^3,4^.

However, compared with open surgery, MAS can be challenging due to the restricted movement of instruments and the use of two-dimensional vision, which make accurate dissection and suturing difficult. As a result, the time it takes surgeons to gain competency in MAS can be longer^5,6^. Surgeons have also reported physical discomfort after performing MAS because of typical laparoscopic instrument design^7–9^.

Compared with conventional MAS, robot-assisted MAS can improve, for some surgeons and procedures, three-dimensional visualization, dexterity, and precision. Challenging suturing and dissection can be made easier by using robotic assistance and may lead to improved surgical outcomes^6,10–12^. Robotic assistance may also reduce the length of hospital stays and operative times, as well as the time for surgeons to develop competency in MAS as seen in other surgical specialties^6,10,11^. Furthermore, one of the most serious complications following esophagectomy, anastomotic leakage, as well as other post-operative complications, remains common with MAS^3,13^.

Following robot-assisted esophagectomy, studies have also shown that patients have reduced intra-operative blood loss, anastomotic leaks, lower rates of post-operative pneumonia, respiratory failure, and morbidity, as well as higher rates of R0 resection compared with open or conventional laparoscopic surgery^14–16^.

**Figure 1:**
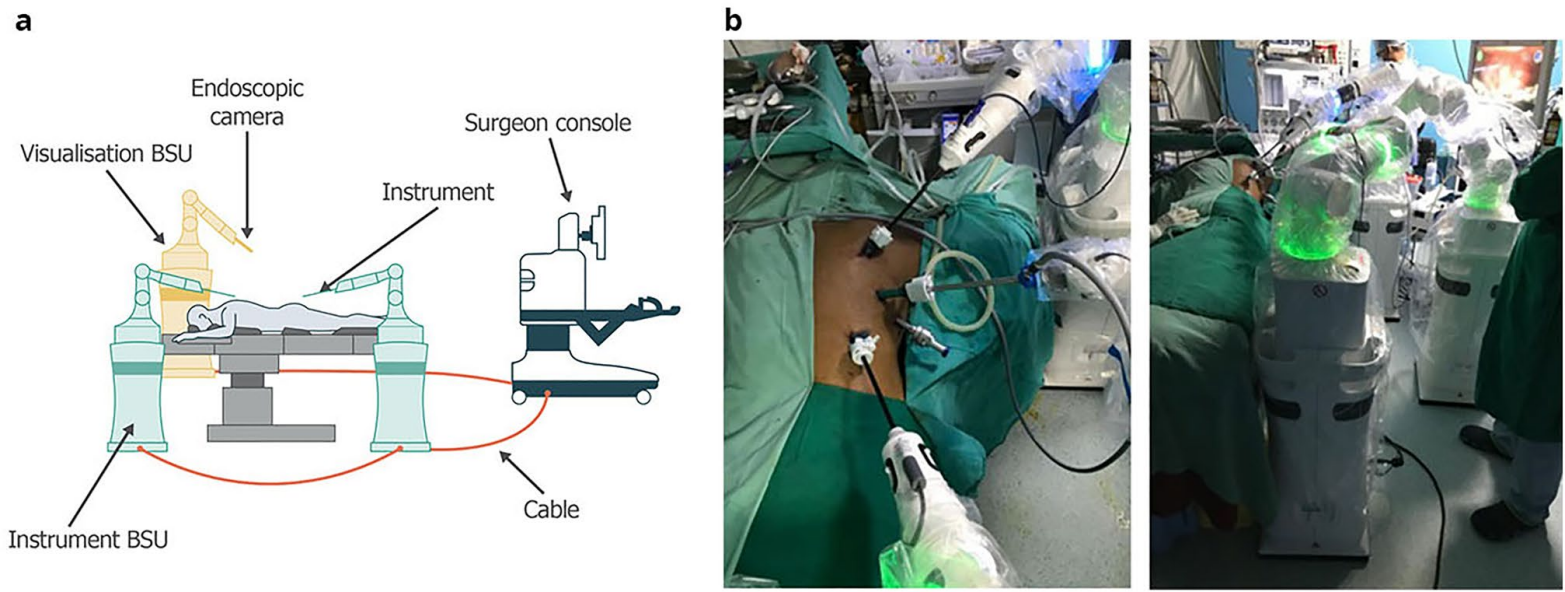
Overview of the Versius Surgical System. Adapted from Haig et al.^17^. Schematic representation of the setup of Versius (**a**) and real-world images of the Versius setup (**b**). BSU: bedside unit.

The Versius Surgical System (CMR Surgical Ltd, Cambridge, UK) is a next-generation tele-operated robotic surgical system designed to assist surgeons in performing MAS (Fig. 1). The surgical system comprises mobile and practically sized bedside units (BSUs), and the ability to vary BSU positions facilitates a greater degree of freedom in port placement, thus allowing the surgeon to replicate a conventional laparoscopic setup. Another distinguishing feature is the eight-jointed robotic arm with a wristed instrument that allows for rotation, pitch and yaw at the end effector. This level of instrument articulation permits the small form that the robot exploits, which may be particularly advantageous for patients with different BMIs. The surgical system is tele-operated in a seated or standing position and features an open console design, minimizing conventional ergonomic challenges without limiting surgeon–surgical team communication. Further, the controller handgrip was based on that of a games console for an optimally ergonomic design^17–19^.

The surgical system has been broadly developed in-line with IDEAL-D (Idea, Development, Exploration, Assessment, Long-term follow-up-Devices) recommendations for generating a sufficient evidence base throughout surgical innovation^20,21^. Initial studies sought to fulfil Stages 0 and 1 (Ideas) to demonstrate proof of concept^17,18,22^. Preclinical evaluation demonstrated that the new system can be used to undertake a range of procedures in cadaver and live porcine studies^19,23–25^.

First-in-human clinical trials have subsequently shown that the surgical system can be used successfully in gynecological and cholecystectomy interventions towards fulfilling Stage 2b (Exploration)^26,27^. We report the first-in-human series of major transthoracic esophageal resections performed using the Versius Surgical System in a single center.

## Methods

Anonymized data were collected on transthoracic esophagectomies (TTEs) performed using the surgical system. All methods were carried out in accordance with the relevant regulations and guidelines, according to ISO14155 standards and informed consent was obtained from all patients. All study protocols and surgical procedures were reviewed and approved by the Institutional Ethics Committee, Galaxy Care Multispecialty Hospital, Pune, Maharashtra, India in August 2019. Data were recorded in a prospectively maintained database, for which written consent was obtained from each patient.

To be eligible for surgery, patients must have been aged ≥ 18 years and, if female and of childbearing potential, patients must not have been pregnant. Patients must have had either a T1T2N0 tumor, localized T3N0 tumor or T3 or T4 tumors that were unresponsive to chemotherapy. Patients were excluded from surgery if they had either a Tracheo-esophageal fistula, metastatic tumor, non-responding tumor to chemotherapy and/or were medically unfit (based on the American Society of Anesthesiologists [ASA] Status; patients with an ASA grade of 4 or 5 were excluded)^28^.

Baseline demographic data were collected along with data describing operation type, indication and body mass index (BMI); ASA Status was also recorded. Pathology data and the number of lymph nodes resected from each patient were also collected. The robotic system’s ability to achieve tumor clearance (measured by R0 resection rates, defined as the absence of residual tumor at 1 mm of the residual margin^14^) whilst reducing anastomotic leakage rates was evaluated. Peri-operative parameters documented were total operating time (from incision to skin closure), estimated intra-operative blood loss, the need for intra-operative blood transfusion, any return to surgery within 24 h and the length of initial hospital stay.

Intra-operative and post-operative complications were also monitored. Post-operative complications were monitored and graded using the Clavien-Dindo classification. Following discharge, patients continued to be monitored for complications or readmission up to 90 days post-surgery and received follow-up in-clinic consultations. Data are presented as continuous data summaries outlining the number of observations, the median, and range.

### Surgical team

All procedures were completed by a single specialist oncosurgeon (S. Puntambekar [SP]) who has extensive experience performing robot-assisted surgery (> 800 cases). Procedures took place at the Galaxy Care Hospital, Pune, Maharashtra, India between 22 August 2019 and 17 June 2021. All surgical team members completed and passed a validated 3.5 day training program^22^. This was in addition to completing a didactic online program and simulated practice using the surgical system.

### System set-up

**Figure 2:**
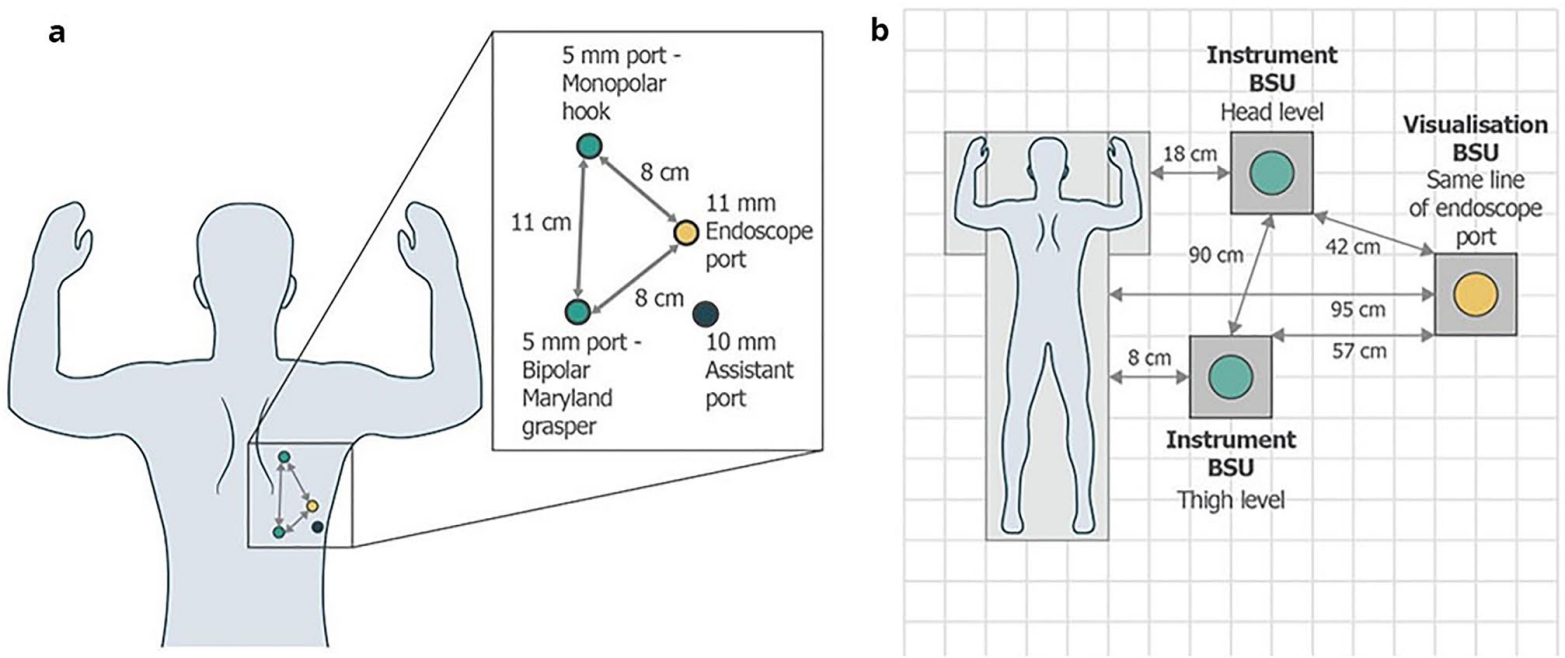
Port positioning and operating room layout. Port positioning for TTE (**a**) with corresponding BSU positions (**b**). An 11 mm endoscope port was placed in the 5th or 6th intercostal space. The right 5 mm instrument port was placed approximately in the 3rd intercostal space. The left 5 mm instrument port was placed in the 7th–8th intercostal space. One 10 mm assistant port was placed between the left instrument port and the endoscope port. *BSU* bedside unit, *TTE* transthoracic esophagectomy.

The robotic system consisted of a surgeon console, two instrument BSUs and one visualization BSU. The surgeon console was orientated such that the surgeon had a clear line of vision to the patient and operating room (OR) team. The 11 mm endoscope port, positioned on the right side of the patient, was set to 0 degrees and placed one finger breadth below and posterior to the inferior angle of the scapula in the 5th or 6th intercostal space. The right 5 mm instrument port was positioned towards head level and placed approximately in the 3rd intercostal space, whereas the left 5 mm instrument port was positioned towards thigh level in the 7th–8th intercostal space. An assistant port was used for clip applicator and suctioning. The two instrument BSUs were positioned on the right side of the patient and on either side of the visualization BSU. The patient port placements and BSU positions within the OR for TTE are presented in Fig. 2.

### Surgical procedures and evaluations

The TTEs were minimal access McKeown’s procedures with cervical esophagogastric anastomosis, performed using a three-hole approach. The surgical system was used only for the thoracic esophagus mobilization phase of the procedures. Each patient was placed in a prone position. The procedure commenced by cutting the inferior pulmonary ligament, which was extended to the right main bronchus. The right vagus nerve was identified and a dissection between the nerve and pericardium was performed. The esophagus was mobilized from the pericardium.

The right main bronchus was then dissected from the esophagus and all subcarinal nodes were removed. An incision to the right vagus nerve was made at the level of the tracheal carina and dissection was performed above the carina between the azygos vein and esophagus. The esophagus was then retracted medially, and dissection was performed between the descending aorta and the esophagus. The direct branches of the aorta supplying the esophagus were bipolarized and dissected until the left main bronchus was mobilized from the esophagus. The left vagus nerve was also dissected and mobilized at the level of carina.

Dissection continued until the crus of diaphragm, taking care to remove all esophageal lymph nodes. The thoracic duct was either clipped or dissected away from the esophagus and the left pleura was avoided, achieving entire infra-azygos mobilization of the esophagus.

The lympho-areolar tissue was then removed along with the esophagus, with care taken to preserve the azygos vein and bronchial artery, and dissection was extended cranially up to the root of the neck. The supra-azygos partial pleura covering the esophagus was cut to allow access to the supra-azygos esophagus, and all paratracheal nodes were removed whilst care was taken to avoid damage to the posterior wall of the trachea.

The gastric mobilisation and gastric pull-up into posterior mediastinum were done laparoscopically in Lloyd-Davis position. The cervical esophagogastric anastomosis was performed in the neck via a left cervical incision, and the anastomosis was completed in two layers with silk 3-0 interrupted sutures; the stomach was used for preparing a conduit in all cases.

## Results

### Patient disposition and baseline characteristics

In total, 80 patients were assessed for surgical eligibility and 57 underwent robot-assisted TTE. Of these, 35.1% were female and the median age was 62 years (range 28–87). The median patient BMI was 24.2 kg/m^2^ (range 14.3–38.5 kg/m^2^). The majority of patients underwent TTE as treatment for malignant neoplasm of the esophagus; 30 patients (52.6%) were treated for squamous cell carcinoma and 26 (45.6%) for adenocarcinoma, whilst one patient (1.8%) was treated for a benign neoplasm (leiomyoma) of the esophagus (Table 1).

**Table 1:** Patient characteristics, surgical history and R0 resection rate.

	Robot-assisted esophagectomy (n = 57), n (%)*
**Characteristic**	
Sex	
Female	20 (35.1)
Age (years), median (range)	62 (28–87)
Height (cm), median (range)	160 (140–173)
Weight (kg), median (range)	62 (35–96)
BMI (kg/m^2^), median (range)	24.2 (14.3–38.5)
< 18.5	4 (7.0)
18.5– < 25	30 (52.6)
25– < 30	20 (35.1)
30– < 40	3 (5.3)
≥ 40	0 (0.0)
ASA status	
Class I	9 (15.8)
Class II	47 (82.5)
Class III	1 (1.8)
**Surgical history**	
Diagnoses	
Squamous cell carcinoma	30 (52.6)
Adenocarcinoma	26 (45.6)
Benign neoplasm (leiomyoma)	1 (1.8)
R0 resection**	
Achieved	57 (100)

*Data are expressed as n (%) unless specified otherwise.

**R0 resection is defined as the absence of residual tumor at 1 mm of the residual margin^14^.

*ASA* American Society of Anesthesiologists^28^, *BMI* body mass index.

### Intra-operative and pathology outcomes

**Figure 3:**
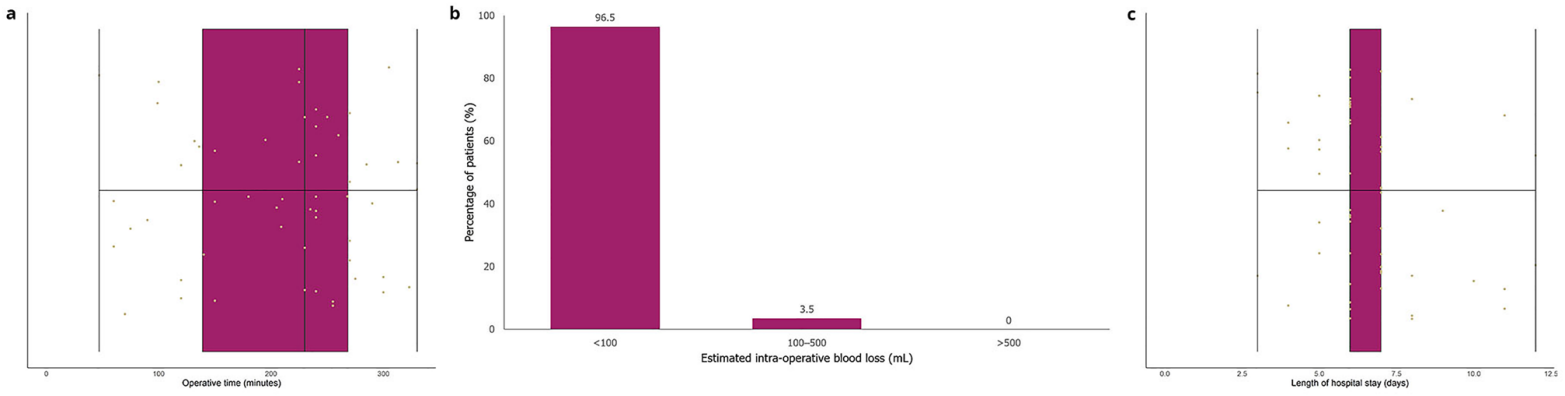
Outcomes of robot-assisted TTE. Operative time from first incision to skin closure (**a**); estimated intra-operative blood loss (**b**); and number of days from operation to discharge (**c**). For (**a**) and (**c**), the middle vertical lines represent the medians, left and right box edges represent the first and third quartiles, and the lower and upper whiskers extend to the respective lowest and highest values.

The TTEs proceeded only once an assessment had confirmed that R0 resection rates could be achieved by adequate macroscopic surgical margins; subsequently, R0 resection was achieved in all TTE procedures. All 57 TTEs were completed using the robotic system with no unplanned conversions to an alternative surgical method. From incision to skin closure, the median operative time was 230 min (range: 47–330 min) (Fig. 3a). Estimated intra-operative blood loss was minimal with no patient losing more than 500 mL of blood (Fig. 3b). There were no intra-operative complications. The number of lymph nodes resected from each patient ranged from 8 to 18, with an average of 14.

### Post-operative outcomes

From the date of the procedure to discharge, the median length of hospitalization was 6 days (range 3–12 days; Fig. 3c). No patients returned to the OR within 24 h of TTE or were readmitted to hospital, and no post-operative complications or deaths were recorded in the 90-day follow-up period.

## Discussion

This report demonstrates the feasibility of a next-generation tele-operated robotic surgical system for use in performing TTE procedures. All 57 robot-assisted surgical procedures were performed successfully without conversion to an alternative surgical method. The estimated intra-operative blood loss was minimal, and no intra-operative or post-operative adverse events, nor patient readmissions or deaths, were reported.

Robotic TTE was used in this patient population to further assess the robotic system’s capability to achieve tumor clearance, measured via R0 resection rates, whilst preserving the azygos vein and reducing anastomotic leakage rates. Robot-assisted MAS is associated with higher rates of R0 resection compared with conventional MAS^14,29^ and open surgery^4^. We provide further evidence to show that R0 resection rates using robot-assisted surgery in patients requiring TTE are comparable to other surgical methods, thus demonstrating the effectiveness of this surgical system.

Furthermore, in conventional MAS, the ligation of the azygos vein is routinely performed to allow access to the esophagus^30^. However, preserving the azygos vein helps prevent reflux and maintains the natural venous system, thus enhancing patient quality of life^30^. Preservation of the vein also prevents post-operative edema by aiding venous drainage, which protects against anastomotic leakage^31^.

Anastomotic leakage is common with MAS and is a major complication following TTE, impacting an individual’s ability to eat and drink^13^. Whilst other studies have reported leakage rates as low as 2.0%^13^, no patient who underwent robot-assisted TTE experienced anastomotic leakage, suggesting that this surgical system may facilitate at least comparable anastomotic leakage rates to current systems when performing TTE.

Furthermore, the median operative time presented in this report was 4 h, which is comparable to times reported by other studies detailing the early use of robotic surgical systems for esophagectomy and may decrease further as surgeons gain experience with the system^32,33^. The dexterity of the surgical system during TTE procedures was comparable to existing market robots, according to the lead author (SP), and the individual robotic arms of the surgical system described herein provided flexibility for port positioning, especially in patients with very low BMIs.

The results described in this report support further assessment in a larger number of patients with different surgical needs. These studies would aim to fulfil IDEAL-D framework Stage 3 (Assessment), by providing evidence of middle- and long-term clinical outcomes^20,21^.

Data collected in this clinical experience study have been entered into the Versius surgical registry, an ongoing collection of real-world data and evidence to evaluate ongoing patient safety. This registry has been designed to fulfil IDEAL-D framework Stage 4 (Long-Term study), by enabling surveillance of rare events, long-term clinical outcomes and quality assurance^20,21^.

## Conclusions

These results show that the Versius Surgical System may be used to perform TTE successfully and safely, highlighting the need for further evaluation.

## Data Availability

Where data can be anonymised, CMR Surgical will share all individual participant data that underlie the results reported in this article with qualified researchers who provide a valid research question. Study documents are available. Proposals should be submitted to mark.slack@cmrsurgical.com and will be assessed by a scientific review board. Raw data are available beginning 6 months and ending 5 years after publication.
